# Lupeol alleviates atopic dermatitis-like skin inflammation in 2,4-dinitrochlorobenzene/*Dermatophagoides farinae* extract-induced mice

**DOI:** 10.1186/s40360-023-00668-9

**Published:** 2023-04-25

**Authors:** Sojung Bae, Na-Hee Jeong, Young-Ae Choi, Byungheon Lee, Yong Hyun Jang, Soyoung Lee, Sang-Hyun Kim

**Affiliations:** 1 CMRI, Department of Pharmacology, School of Medicine, Kyungpook National University, Daegu, Republic of Korea; 2 Department of Biochemistry and Cell Biology, School of Medicine, Kyungpook National University, 680 Gukchaebosang-ro, Jung-gu, Daegu, 41944 Republic of Korea; 3 Department of Dermatology, School of Medicine, Kyungpook National University Hospital, 130 Dongdeok-ro, Jung-gu, Daegu, 41944 Republic of Korea; 4 Immunoregulatory Materials Research Center, Korea Research Institute of Bioscience and Biotechnology (KRIBB), 181 Ipsin-gil, Jeongeup, 56212 Republic of Korea

**Keywords:** Atopic dermatitis, House dust mite, Keratinocytes, Lupeol, Skin inflammation

## Abstract

**Background:**

Atopic dermatitis (AD) is a chronic inflammatory skin disease that affects from children to adults widely, presenting symptoms such as pruritus, erythema, scaling, and dryness. Lupeol, a pentacyclic triterpenoid, has anti-inflammatory and antimicrobial activities. Based on these properties, the therapeutic effects of lupeol on skin disorders have been actively studied. In the present study, we aimed to determine the effectiveness of lupeol on AD.

**Methods:**

We utilized tumor necrosis factor (TNF)-α/interferon (IFN)-γ-stimulated keratinocytes and 2, 4-dinitrochlorobenzene/*Dermatophagoides farinae* extract (DFE)-induced AD mice to confirm the action.

**Results:**

Lupeol inhibited TNF-α/IFN-γ-stimulated keratinocytes activation by reducing the expressions of pro-inflammatory cytokines and chemokines which are mediated by the activation of signaling molecules such as signal transducer and activator of transcription 1, mitogen-activated protein kinases (p38 and ERK), and nuclear factor-κB. Oral administration of lupeol suppressed epidermal and dermal thickening and immune cell infiltration in ear tissue. Immunoglobulin (Ig) E (total and DFE-specific) and IgG2a levels in serum were also reduced by lupeol. The gene expression and protein secretion of T helper (Th) 2 cytokines, Th1 cytokines, and pro-inflammatory cytokine in ear tissue were decreased by lupeol.

**Conclusions:**

These results suggest that lupeol has inhibitory effects on AD-related responses. Therefore, lupeol could be a promising therapeutic agent for AD.

**Supplementary Information:**

The online version contains supplementary material available at 10.1186/s40360-023-00668-9.

## Background

Atopic dermatitis (AD) is a skin inflammatory disease with an increasing prevalence worldwide [1]. AD usually develops in early childhood and lasts into adulthood or sometimes begins in adulthood [2, 3]. It is the most common skin disorder that imposes a significant burden on patients’ lives [4]. Furthermore, several studies have shown that AD gives rise to various allergic diseases such as asthma and allergic rhinitis in the majority of afflicted patients [5].

The pathogenesis of AD is associated with skin barrier dysfunction and abnormal immune response based on genetic, environmental, and psychological factors [6]. Skin barrier dysfunction caused by multiple factors enables the entry of allergens like house dust mites (HDMs) [7, 8]. Activated keratinocytes produce pro-inflammatory cytokines like interleukin (IL)-1β and IL-6 and chemokines like CC chemokine ligand (CCL) 17 and CCL22, which attract immune cells into skin lesions and aggravate AD [9, 10]. In the acute phase of AD, allergens primarily induce T helper (Th) 2 cell immune response like secretion of cytokines such as IL-4, IL-5, and IL-13 which lead to immunoglobulin (Ig) E class switching and immune cell infiltration [11]. In addition, Th2-related cytokines modulate filaggrin expression, resulting in skin barrier defects [12]. When AD progresses to the chronic phase, Th1 response is also increased with the secretion of interferon (IFN)-γ [13]. Immune cells recruited into skin lesions secrete inflammatory mediators that activate keratinocytes and exacerbate inflammatory responses [14]. Accordingly, regulating the inflammatory responses of keratinocytes and immune cells is the main therapeutic approach for AD.

In common, glucocorticosteroids, calcineurin inhibitors, and antihistamines are used as therapeutic agents for AD [15]. Continuous use, on the other hand, can cause a range of side effects, including skin atrophy, gastritis, adrenal insufficiency, weight gain, and emotional lability [16]. Therefore, developing an alternative agent that exhibits both therapeutic effect and safety is urgently necessary.

Lupeol, a naturally occurring triterpene found in various fruits, vegetables, and medicinal plants, possesses a broad spectrum of pharmacological activities against inflammation, allergy, microbial activities, oxidative stress, and cancer [17–19]. Lupeol has been proven to inhibit various inflammatory responses in the 12-O-tetradecanoylphorbol-13-acetate-induced inflammation [20]. A previous study also reported that lupeol has therapeutic potential on asthma, a chronic inflammatory disease associated with Th2 immune response [21]. In addition, the effects of lupeol on skin disorders such as acne, wound, and melanoma have been recently studied based on their pharmacological activities [22–24]. Although lupeol exhibits various therapeutic effects on inflammatory diseases and skin disorders, its effectiveness on AD-like skin inflammation has not been identified yet. In this study, we concentrated on the applicability of lupeol as a therapeutic agent for AD based on its known properties. Based on our study, we could expand the range of pharmacological activities of lupeol specifically as an alternative therapeutic candidate for AD.

## Methods

### Reagents

**Fig. 1:**
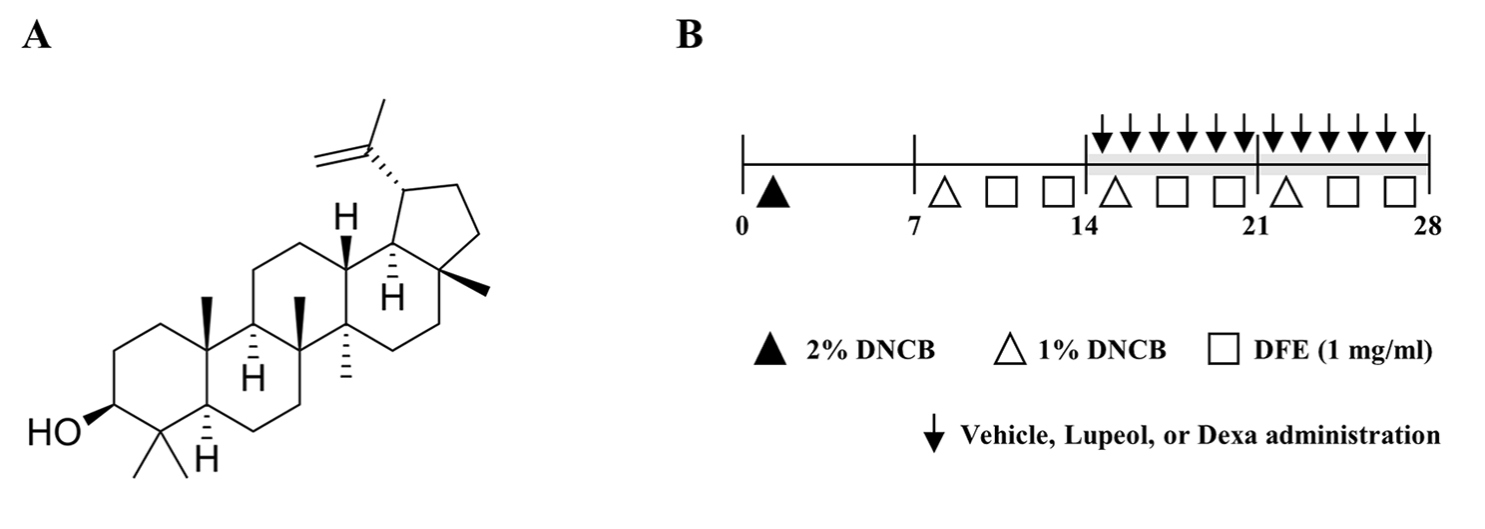
Chemical structure of lupeol and the experimental scheme of AD induction. (A) The chemical structure of lupeol. (B) Experimental design of the study. Both ears were painted with 2% DNCB (20 µl/each ear) for sensitization in the first week. Both ears were then applied 1% DNCB (20 µl/each ear) once and 1 mg/ml DFE (20 µl/each ear) twice a week for 3 weeks. After two weeks of induction, vehicle, lupeol or Dexa was administered orally 6 times a week

Lupeol (Sigma-Aldrich, St. Louis, MO, USA) was dissolved in dimethyl sulfoxide (DMSO) for cell experiments and in phosphate-buffered saline (PBS) with 0.5% sodium carboxymethyl cellulose for animal experiments. Figure 1 A depicts the chemical structure of lupeol. 2, 4-dinitrochlorobenzene (DNCB) was dissolved in a mixture of acetone and olive oil (3:1), and *Dermatophagoides farinae* extract (DFE, Prolagen, Seoul, Republic of Korea) was dissolved in PBS with 0.5% Tween 20. All the other reagents were purchased from Sigma-Aldrich unless otherwise stated.

### Cell culture

HaCaT (CLS Cell Lines Service), a human keratinocyte cell line, was cultured in Dulbecco’s modified Eagle medium (Gibco, Grand Island, NY, USA) containing 10% heat-inactivated fetal bovine serum (Gibco), 100 U/ml of penicillin G, and 100 µg/ml of streptomycin in 5% CO_2_ at 37 °C. Recombinant tumor necrosis factor (TNF)-α and IFN-γ were purchased from R&D Systems (Minneapolis, MN, USA) to stimulate HaCaT cells.

### Cell viability

Cell viability was measured using MTT (3-(4,5-dimethylthiazol-2-yl)-2,5-diphenyltetrazolium bromide) assay. HaCaT cells were seeded in a density of 1 × 10^4^ cells per well in a 96-well plate and stabilized overnight. Then, HaCaT cells were incubated with each concentration of lupeol (0-100 µM) or DMSO (0.5%) for 24 h. Subsequently, 20 µl of MTT solution (5 mg/ml in PBS) was added to each well, and the cells were incubated for 4 h. The formazan crystals were dissolved in 100 µl of DMSO, and the absorbance was measured using a spectrophotometer (VersaMax™ Microplate Reader, Molecular Devices, San Jose, CA, USA) at 570 nm. Cell viability was determined in comparison to the non-treated group (100%).

### Animals

BALB/c mice (female, 4 weeks old, *n* = 35) were supplied from Dae-Han Experimental Animal Center (Daejeon, Republic of Korea) and maintained per cage with five mice in a breeding room. The room was kept at a constant temperature of 22 ± 2 °C, relative humidity of 55 ± 5%, and provided a 12 h light/dark cycle. Prior to the start of the experiment, the mice were maintained for one week. The animal study was carried out in accordance with ARRIVE guidelines and Public Health Service Policy on the Humane Care and Use of Laboratory Animals guidelines. The experiment was approved by the Institutional Animal Care and Use Committee of Kyungpook National University (IRB # 2021-0073).

### Induction of AD-like skin inflammation

To induce AD-like skin inflammation, we followed the steps outlined in Fig. 1B. The mice were randomly assigned to one of the seven groups (*n* = 5/group): Group I. vehicle (PBS with 0.5% sodium carboxymethyl cellulose), Group II. lupeol 10 mg/kg, Group III. DNCB/DFE plus vehicle, Group IV. DNCB/DFE plus lupeol 0.1 mg/kg, Group V. DNCB/DFE plus lupeol 1 mg/kg, Group VI. DNCB/DFE plus lupeol 10 mg/kg, and Group VII. DNCB/DFE plus dexamethasone (Dexa) 1 mg/kg. In this study, Dexa was used as a positive drug control. In the first week, both ears were painted with 2% DNCB (20 µl/each ear) for sensitization. Both ears were then treated with 1% DNCB (20 µl/each ear) once and 1 mg/ml DFE (20 µl/each ear) twice a week for 3 weeks. Vehicle, lupeol, or Dexa was administered orally six times per week after two weeks of induction. After 24 h from DNCB/DFE application, ear thickness was measured using a dial gauge (Mitutoyo, Tokyo, Japan). On day 28, the mice were euthanized by CO_2_, and whole blood samples were obtained from the abdominal aorta. The blood samples were centrifuged for 15 min at 1,000 × *g* after 2 h of coagulation, and then serum was collected. The mouse ears were excised for histological observation, RT-*q*PCR, and ELISA. The liver and kidney were collected to confirm toxicity.

### RT-***q***PCR

Total RNA was isolated from HaCaT cells and mouse ear tissues with an RNAiso Plus kit (Takara Bio, Shiga, Japan). HaCaT cells seeded in a density of 2 × 10^5^ cells per well in a 24-well plate were treated with lupeol (0.1, 1, or 10 µM) or Dexa (10 µM). After 2 h, HaCaT cells were stimulated with 10 ng/ml of TNF-α and IFN-γ for 6 h. The ear tissues were homogenized using a Tissue Lyser II (Qiagen, Hilden, Germany). Total RNA from the samples was quantified using a NanoDrop 2000 spectrophotometer (Thermo Fisher Scientific, Waltham, MA, USA), and then cDNA was synthesized using a RevertAid RT Kit (Thermo Fisher Scientific). Subsequently, RT-*q*PCR was performed using a StepOnePlus real-time PCR system (Thermo Fisher Scientific) with 0.3 µM of each primer (shown in Table 1) and 2X QGreenBlue Master mix (Cellsafe, Yong-in, Republic of Korea). The amplification conditions were as follows: 3 min at 95 °C, 40 cycles of 10 s at 95 °C, 30 s at 60 °C, and then melting curve analysis was done. The gene expression was normalized to glyceraldehyde 3-phosphate dehydrogenase (GAPDH), and the analysis was performed using the StepOnePlus PCR system software v2.3.

**Table 1:** Sequences of the primer pairs used for RT-*q*PCR

Mouse		
Primer	Sequence 5’→3’	GenBank accession number
IFN-γ	F: GCCACGGCACAGTCATTGA R: TGCTGATGGCCTGATTGTCTT	NM_008337.4
IL-1β	F: GGACCTTCCAGGATGAGGAC R: GTTCATCTCGGAGCCTGTAG	NM_008361.4
IL-4	F: ATCATCGGCATTTTGAACGAGGTC R: ACCTTGGAAGCCCTACAGACGA	NM_021283.2
IL-5	F: GAAGTGTGGCGAGGAGAGAC R: GCACAGTTTTGTGGGGTTTT	NM_010558.1
IL-12 A	F: GATGACATGGTGAAGACGGC R: AGGCACAGGGTCATCATCAA	NM_008351.3
IL-13	F: GGGACATGGTTTGCTGCCTA R: AGACAGGAGTGTTGCTCTGG	NM_008355.3
GAPDH	F: TGCTCCTCCCTGTTCCAGA R: TACGGCCAAATCCGTTCACA	NM_008084.3
**Human**		
Primer	Sequence 5’→3’	GenBank accession number
CCL17	F: ACAAGGGGATGGGATCTCCCTCAC R: ACTGCTCCAGGGATGCCATCGTTT	NM_002987.3
CCL22	F: AGGACAGAGCATGGATCGCCTACA R: TAATGGCAGGGAGGTAGGGCTCCT	NM_002990.5
IL-1β	F: GCTGATGGCCCTAAACAGATGAA R: TGAAGCCCTTGCTGTAGTGGTG	NM_000576.3
IL-6	F: CCCCTGACCCAACCACAAAT R: CATTTGCCGAAGAGCCCTCA	NM_000600.5
GAPDH	F: AGACACCATGGGGAAGGTGA R: TGGAATTTGCCATGGGTGGA	NM_002046.7

F, forward; R, reverse

### ELISA

Using ELISA kits (R&D Systems), the levels of secreted IL-1β, IL-6, CCL17, and CCL22 were measured in conditioned media of stimulated HaCaT cells. HaCaT cells seeded in a density of 2 × 10^5^ cells per well in a 24-well plate were treated with lupeol (0.1, 1, or 10 µM) or Dexa (10 µM). After 2 h, HaCaT cells were stimulated with 10 ng/ml of TNF-α and IFN-γ for 24 h. The supernatants were centrifuged at 1,000 × *g* for 5 min at 4 °C. The samples were then analyzed following the manufacturer’s protocol.

IgE (total and DFE-specific) and IgG2a levels in serum were measured using ELISA kits according to the manufacturer’s instructions (BD Biosciences, Franklin Lakes, NJ, USA). To detect DFE-specific IgE, wells were pre-coated with 30 µg/ml of DFE in PBS, and the following steps were carried out according to the manufacturer’s instructions for IgE.

Ear tissues were homogenized with an extraction buffer (100 mM Tris; pH 7.4, 150 mM NaCl, 1 mM EGTA, 1 mM EDTA, 1% Triton X-100, and 0.5% sodium deoxycholate) added with protease inhibitor cocktail (Roche, Basel, Switzerland) and 1 mM of phenyl methyl sulfonyl fluoride using the Tissue Lyser II. Homogenates were centrifuged for 15 min at 18,000 × *g* to remove debris. The protein levels of IL-4 (BD Biosciences), IFN-γ, and IL-1β (Invitrogen, Waltham, MA, USA) in the supernatant of ear tissue homogenates were detected by each ELISA kit. The absorbance was determined at 450 nm using the spectrophotometer.

### Western blot

HaCaT cells seeded in a density of 1 × 10^6^ cells per well in a 6-well plate were treated with lupeol (10 µM) or Dexa (10 µM). After 2 h, HaCaT cells were stimulated with 10 ng/ml of TNF-α and IFN-γ for 15 min. Total protein extracts were prepared using a lysis buffer (20 mM Tris, 137 mM NaCl, 2 mM EDTA, 10% glycerol, 1% Triton X-100, 0.5 mM DTT, 0.5 mM PMSF, and 1 mM Na_3_VO_4_) supplemented with protease and phosphatase inhibitor cocktails (Roche). The lysate was sonicated for 60 s and centrifuged at 18,000 × *g* for 20 min at 4 °C. Total proteins were collected from the supernatants. Cytosolic and nuclear proteins were fractionated with each buffer. First, cells were collected with a cytosolic lysis buffer (150 mM NaCl, 10 mM HEPES, 1 mM EDTA, 0.5 mM DTT, 0.5 mM PMSF, and 0.5% Triton X-100) added with a protease inhibitor cocktail. After the lysate was centrifuged at 2,500 × *g* for 5 min at 4 °C, cytosolic proteins were collected from the supernatants. The remained pellets were then washed with PBS before being suspended in a nuclear lysis buffer (420 mM NaCl, 20 mM HEPES, 0.2 mM EDTA, 1.2 mM MgCl_2_, 0.5 mM DTT, 0.5 mM PMSF, and 25% glycerol). The suspended pellets were sonicated and centrifuged at 18,000 × *g* for 20 min at 4 °C. Nuclear proteins were collected from the supernatants. Proteins were quantified using a Bradford assay kit (Bio-Rad, Hercules, CA, USA). The protein samples derived from the same experiment were then electrophoresed by 10% sodium dodecyl sulfate-polyacrylamide gel and transferred to nitrocellulose membrane (Pall Life Sciences, Port Washington, NY, USA) in parallel. The membranes were blocked with blocking buffer (4% bovine serum albumin in Tris-buffered saline with 0.1% Tween 20) for 1 h at room temperature and incubated with primary antibody overnight at 4 °C. Then, the membranes were incubated with secondary antibody for 1 h at room temperature. The antibodies used in the experiment are shown in Table 2. The detection was performed using G: BOX Chemi XRQ (Syngene, Cambridge, UK) with a SuperSignal West Pico PLUS chemiluminescent substrate (Thermo Fisher Scientific). The densitometric analysis was conducted using Image J software v1.8.0, and the blots used for the analysis are shown in Additional file 1.

**Table 2:** Antibodies used for Western blot

Target	Supplier	Cat No	Size (kDa)	Host	Dilution
α-Tubulin	Cell Signaling	2144 S	52	Rabbit	1:2000
ERK	Cell Signaling	9102 S	42, 44	Rabbit	1:2000
phospho-ERK^Thr202/Tyr204^	Cell Signaling	9101 S	42, 44	Rabbit	1:2000
IκBα	Santa Cruz	sc-1643	37	Mouse	1:1000
NF-κB p65	Cell Signaling	8242 S	65	Rabbit	1:1000
p38	Cell Signaling	9212 S	40	Rabbit	1:2000
phospho-p38^Thr180/Tyr182^	Cell Signaling	9215 S	43	Rabbit	1:2000
STAT1	Cell Signaling	9172 S	84, 91	Rabbit	1:2000
phospho-STAT1^Tyr701^	Cell Signaling	9167 S	84, 91	Rabbit	1:2000
TBP	Santa Cruz	sc-74596	43	Mouse	1:1000
Mouse IgG	Cell Signaling	7076 S		Horse	1:2000
Rabbit IgG	Cell Signaling	7074 S		Goat	1:2000

### Histological analysis

Mouse ears, liver, and kidney were fixed in a 10% formalin solution for histological analysis. The tissues were embedded in paraffin and cut into a thickness of 5 μm. The sections were stained with hematoxylin and eosin (H&E) for 4 and 2 min respectively and toluidine blue for 1 min 30 s. The thickness of epidermal and dermal layers was measured under ×200 magnification at five randomly selected sites in H&E-stained sections. The number of infiltrated eosinophils and mast cells in ear tissues was counted under ×400 magnification at five random areas in H&E- and toluidine blue-stained sections respectively. The toxicity in organs was confirmed using the sections of the liver and kidney stained with H&E under ×200 magnification.

### Immunohistochemistry (IHC)

Ear tissue sections were deparaffinized using xylene. Then, the sections were rehydrated with graded ethanol solutions and rinsed with deionized H_2_O. Antigen retrieval was produced with Tris-EDTA buffer (10 mM Tris Base, 1 mM EDTA solution, 0.05% Tween 20, pH 9.0) using the microwave for 15 min. To eliminate endogenous peroxidase activity, the sections were treated with 3% hydrogen peroxide for 10 min. The sections were blocked with blocking buffer (5% normal goat serum in PBS with 0.05% Tween 20) for 1 h at room temperature and then incubated with primary antibody against CD4 (1:500, Abcam, Cambridge, UK) overnight at 4℃. Next, biotinylated secondary antibody (1:200, Vector Laboratories, Newark, CA, USA) was applied for 30 min, and ABC-AP (Vector Laboratories) reagent was applied for 30 min. Subsequently, the sections were incubated with AP substrate for 20 min and counterstained with hematoxylin for 1 min at room temperature. IHC-stained sections were observed under ×400 magnification.

### Statistical analysis

Prism 7 (GraphPad Software Inc., La Jolla, CA, USA) was used to conduct the statistical analysis. Results were presented as the mean ± standard error of the mean (SEM). Treatment effects were evaluated using one-way ANOVA with Dunnett’s multiple comparison test. *p* < 0.05 was considered to indicate statistical significance.

## Results

### Effects of lupeol on the expression of pro-inflammatory cytokines and chemokines in TNF-α/IFN-γ-stimulated keratinocytes

**Fig. 2:**
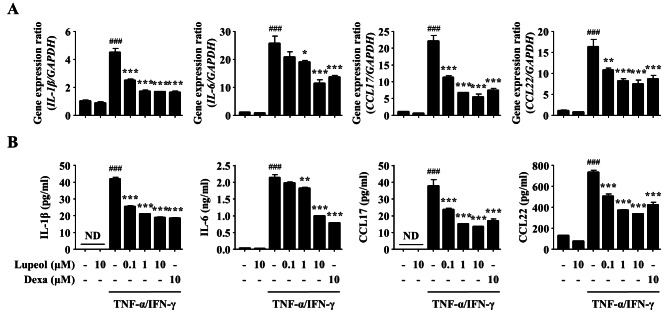
Effects of lupeol on the expression of pro-inflammatory cytokines and chemokines in TNF-α/IFN-γ-stimulated keratinocytes. (A) The gene expression of cytokines and chemokines was determined by RT-*q*PCR. HaCaT cells (2 × 10^5^ cells/well in a 24-well plate) were pretreated with lupeol (0.1, 1, or 10 µM) or Dexa (10 µM) for 2 h, and then stimulated with 10 ng/ml of TNF-α and IFN-γ for 6 h. The gene expression was normalized to GAPDH. (B) The protein secretion was measured by ELISA. HaCaT cells (2 × 10^5^ cells/well in a 24-well plate) were pretreated with lupeol (0.1, 1, or 10 µM) or Dexa (10 µM) for 2 h, and then stimulated with 10 ng/ml of TNF-α and IFN-γ for 24 h. Data are presented as the mean ± SEM (*n* = 3). ^###^*p* < 0.001 versus CON group, *p < 0.05, **p < 0.01, and ***p < 0.001 versus TNF-α/IFN-γ-stimulated group. CON, control; ND, not detected

In AD lesions, activated keratinocytes exacerbate AD pathogenesis by releasing inflammatory mediators such as pro-inflammatory cytokines and chemokines [9]. Therefore, TNF-α/IFN-γ-stimulated HaCaT cells were used to confirm the effects of lupeol on activated keratinocytes. First, the cell viability of lupeol was verified using MTT assay. Lupeol showed no toxicity up to 10 µM in HaCaT cells (Additional file 2A). Subsequently, the gene expression and secretion of pro-inflammatory cytokines (IL-1β and IL-6) and chemokines (CCL17 and CCL22) were analyzed using RT-*q*PCR and ELISA respectively. The gene expression levels of IL-1β, IL-6, CCL17, and CCL22 were increased by TNF-α/IFN-γ stimulation. On the other hand, the pretreatment of lupeol decreased the increased values in a concentration-dependent manner, and these effects were close to the effects of Dexa treatment (Fig. 2A). The secretion of pro-inflammatory cytokines and chemokines also showed a similar trend with gene expression (Fig. 2B).

### Effects of lupeol on the activation of signaling molecules in TNF-α/IFN-γ-stimulated keratinocytes

**Fig. 3:**
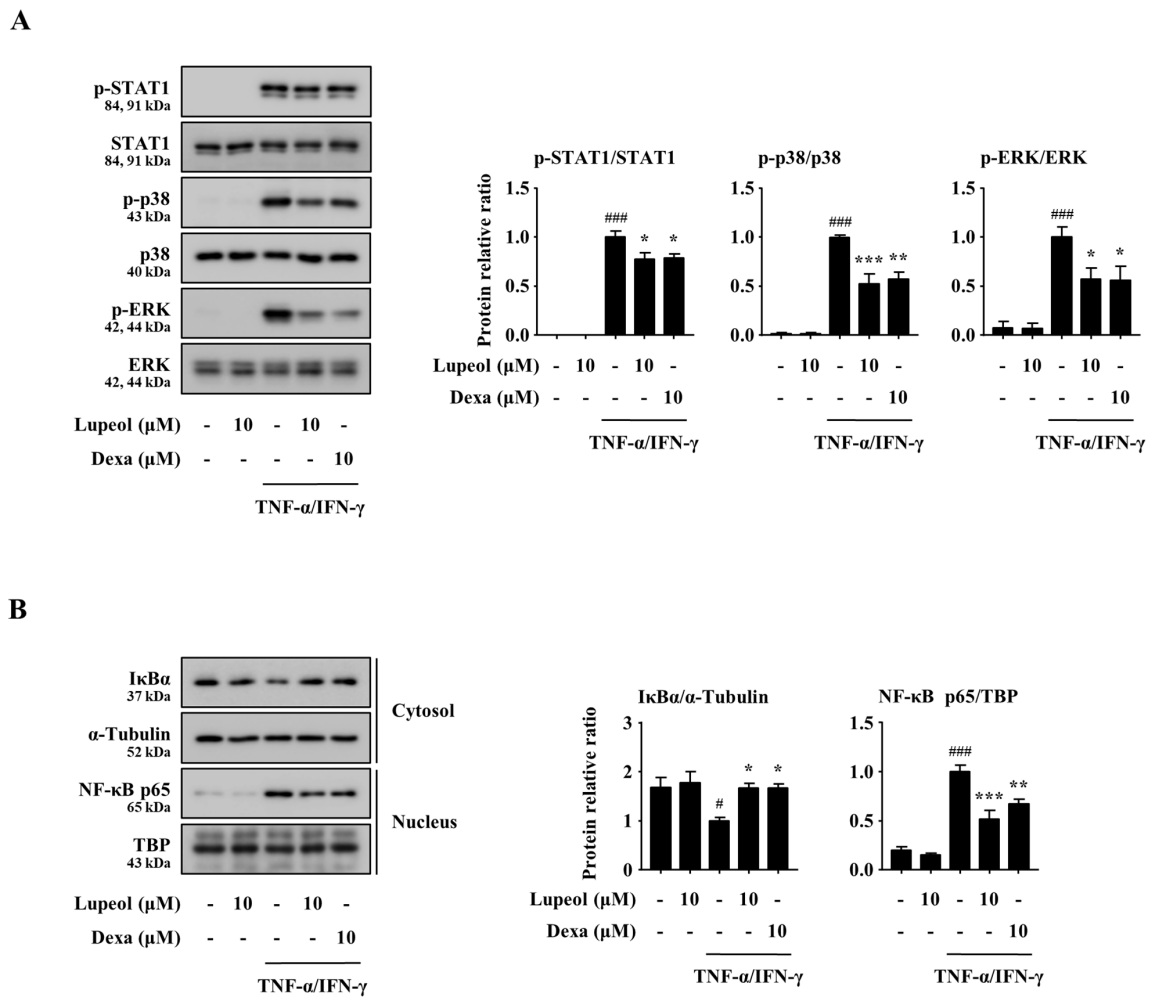
Effects of lupeol on the activation of STAT1, MAPKs, and NF-κB in TNF-α/IFN-γ-stimulated keratinocytes. (A) The phosphorylation of STAT1, MAPKs (p38 and ERK) and (B) the nuclear translocation of NF-κB p65 were detected by Western blot. HaCaT cells (1 × 10^6^ cells/well in a 6-well plate) were treated with lupeol (10 µM) or Dexa (10 µM) for 2 h, and then stimulated with 10 ng/ml of TNF-α and IFN-γ for 15 min. The samples were derived from the same experiment, and gels/blots were processed in parallel. Total form, α-Tubulin, and TBP were used as loading controls. Each blot is a representative result of three independent experiments. All original blots are shown in Additional file 1. The band intensity was measured using Image J software, and the protein relative ratio was calculated in comparison to the TNF-α/IFN-γ-stimulated group. Data are presented as the mean ± SEM (*n* = 3). ^#^*p* < 0.05 and ^###^*p* < 0.001 versus CON group, *p < 0.05, **p < 0.01, and ***p < 0.001 versus TNF-α/IFN-γ-stimulated group. CON, control; p-, phosphorylated

The production of various inflammatory mediators, including IL-1β, IL-6, CCL17, and CCL22, is regulated by signaling molecules such as signal transducer and activator of transcription 1 (STAT1), mitogen-activated protein kinases (MAPKs), and nuclear factor (NF)-κB [25–27]. Thus, we investigated the regulatory mechanisms of lupeol in the expression of inflammatory mediators. TNF-α/IFN-γ treatment in HaCaT cells significantly increased the phosphorylation of STAT1, MAPKs (p38 and ERK) and the nuclear translocation of NF-κB, however, lupeol suppressed the phosphorylation (Fig. 3A) and the nuclear translocation (Fig. 3B). In addition, the signaling molecules were not activated in lupeol only (10 µM) treated group like the control group. These results demonstrate that lupeol reduced the expression of the pro-inflammatory cytokines and chemokines through the blocking of the signaling molecules in keratinocytes.

### Effects of lupeol on the phenotypes in AD-induced mice

**Fig. 4:**
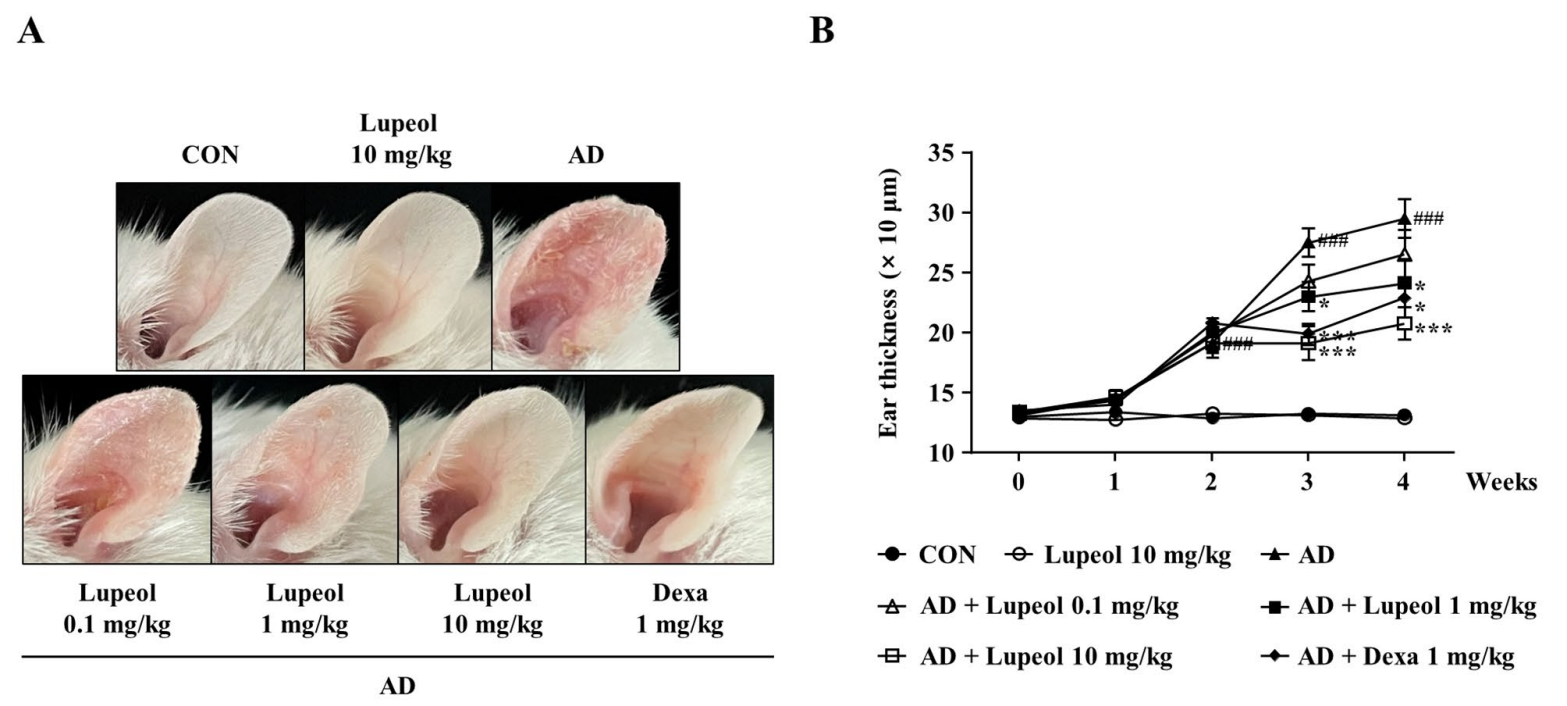
Effects of lupeol on the phenotypes in AD-induced mice. (A) Representative photographs of AD lesions in each experimental mouse group. (B) Ear thickness was measured using a dial gauge 24 h after DNCB/DFE application. Data are presented as the mean ± SEM (*n* = 5). ^###^*p* < 0.001 versus CON group, *p < 0.05 and ***p < 0.001 versus AD-induced group. CON, control

Erythema, scaling, edema, and lichenification are common phenotypes of AD [28]. The effects of lupeol on AD phenotypes were investigated using an AD mouse model created according to the indicated experimental scheme (Fig. 1B). After 2 weeks of induction with DNCB and DFE, vehicle, lupeol (0.1, 1, or 10 mg/kg), or Dexa (1 mg/kg) was administered orally. During the experiment period, the body weights of lupeol administered group did not display significant changes (Additional file 2B). Besides, oral administration of lupeol (10 mg/kg) did not cause changes in organ weight and histological analysis in the liver and kidney (Additional file 2 C and D), indicating that lupeol had no toxicity up to 10 mg/kg. As DNCB/DFE application was continued, AD phenotypes such as erythema, scaling, and edema were getting worse. However, lupeol relieved these phenotypes in a dose-dependent manner (Fig. 4A). The increased ear thickness was considerably recovered from 1 mg/kg lupeol group and more in 10 mg/kg group (Fig. 4B). These results indicate that lupeol alleviated AD phenotypes, including erythema, scaling, and edema.

### Effects of lupeol on the histological alterations in AD-induced mice

**Fig. 5:**
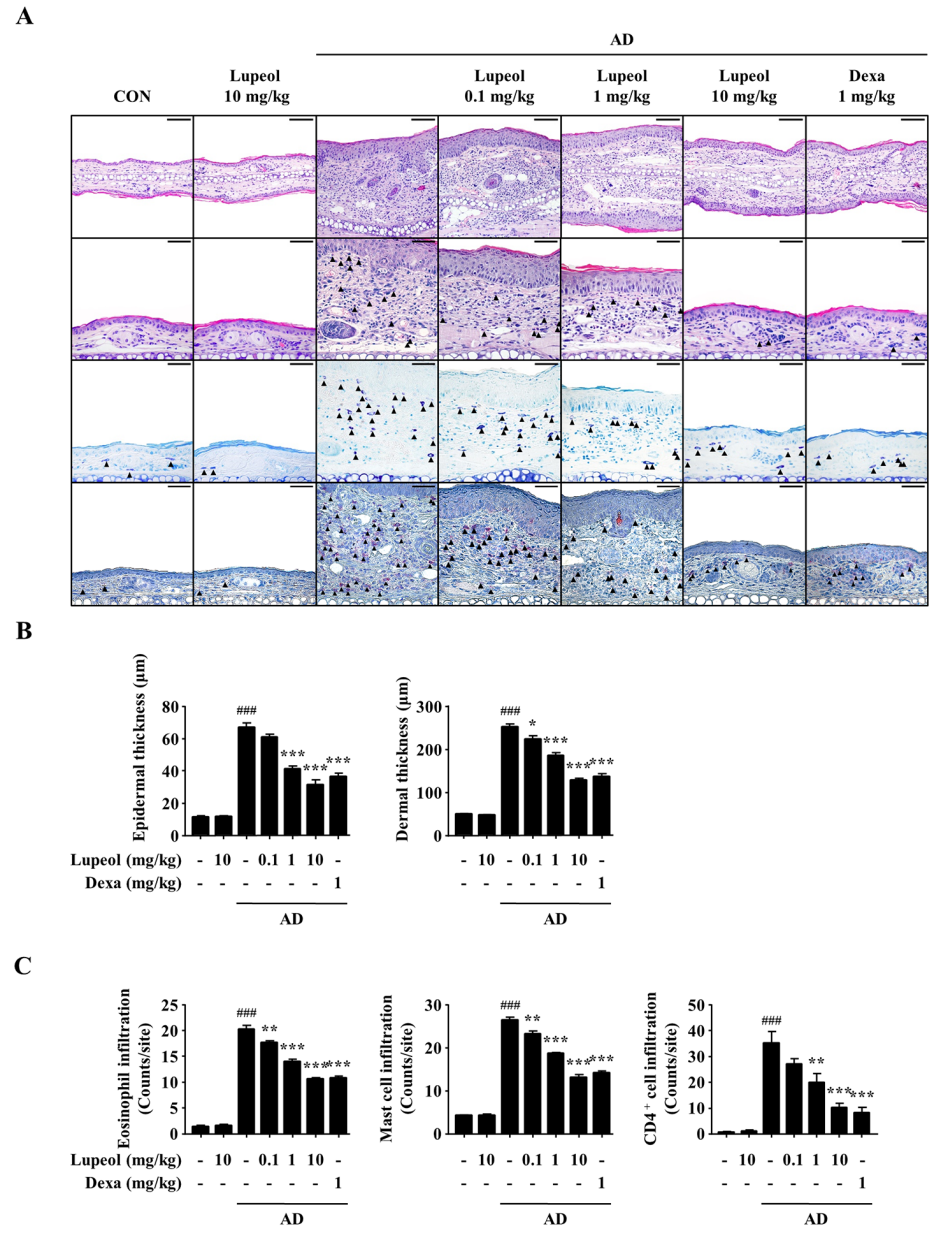
Effects of lupeol on the histological alterations in AD-induced mice. (A) Representative photomicrographs of H&E-stained ear tissues at ×200 magnification (1st panel, scale bar: 100 μm). Representative photomicrographs of ear tissues stained with H&E (2nd panel), toluidine blue (3rd panel), and CD4 antibody (4th panel) at ×400 magnification (scale bar: 50 μm). Each immune cell is indicated by an arrow. (B) Epidermal and dermal thickness was determined in the H&E-stained tissue slides using a micrometer. (C) The number of eosinophils, mast cells, and CD4^+^ immune cells infiltrated into the dermal layer was counted in H&E-, toluidine blue-, and CD4 antibody-stained slides respectively. Data are presented as the mean ± SEM (*n* = 5). ^###^*p* < 0.001 versus CON group, *p < 0.05, **p < 0.01, and ***p < 0.001 versus AD-induced group. CON, control

In histological observation, AD lesions are characterized by increased epidermal and dermal thickness and immune cell infiltration to the dermal layer [29–31]. Thus, the effects of lupeol on histological changes were analyzed in mouse ear tissue. The thickness of epidermal and dermal layers was determined by H&E staining. The increased thickness of the layers was decreased by the oral administration of lupeol similar to the effect on ear thickness (Fig. 5A 1st panel and B). Eosinophil infiltration was counted by H&E staining (Fig. 5A 2nd panel and C), and mast cell infiltration was counted by toluidine blue staining (Fig. 5A 3rd panel and C). Furthermore, CD4^+^ immune cell infiltration was observed by IHC staining (Fig. 5A 4th panel and C). Immune cells infiltrated into the dermal layer were increased by DNCB/DFE induction. However, the number of immune cells, including eosinophils, mast cells, and CD4^+^ immune cells was significantly decreased by lupeol. As a result, lupeol attenuated the histological alterations, including epidermal and dermal thickening and immune cell infiltration in an AD mouse model.

**Fig. 6:**
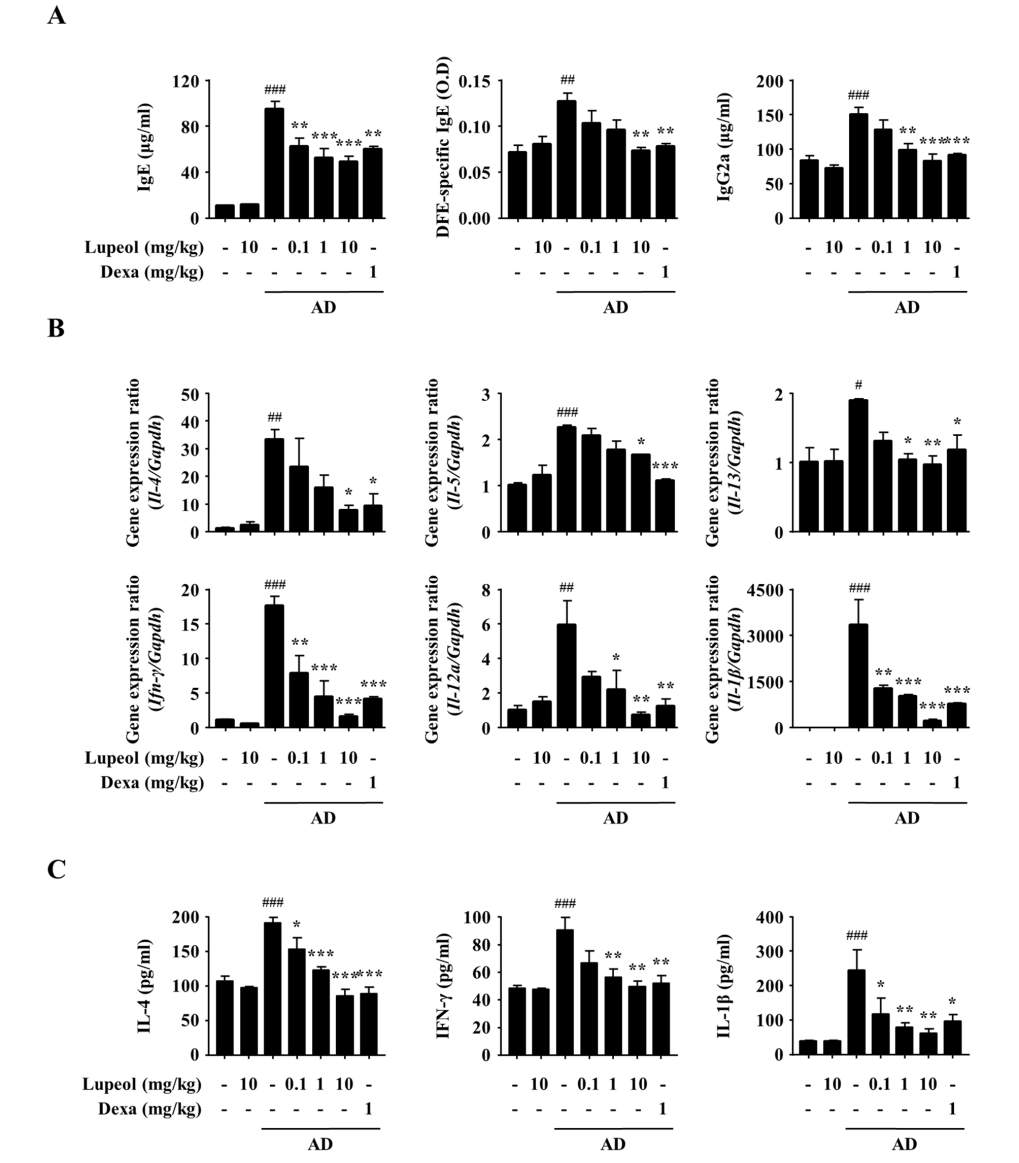
Effects of lupeol on the immunoglobulin and cytokine expression in AD-induced mice. (A) Blood was obtained from the abdominal aorta of each group of experimental mice after they were sacrificed. The serum levels of total IgE, DFE-specific IgE, and IgG2a were measured using ELISA. (B) Both sides of ears were collected from the experimental mice. RNA was isolated from the ears with an RNAiso Plus kit and synthesized into cDNA. The gene expression was measured by RT-*q*PCR. (C) Mouse ears were homogenized with a tissue extraction buffer, and the cytokine levels in the supernatants of the ear tissue homogenates were determined by ELISA. Data are presented as the mean ± SEM (*n* = 5). ^#^*p* < 0.05, ^##^*p* < 0.01, and ^###^*p* < 0.001 versus CON group, *p < 0.05, **p < 0.01, and ***p < 0.001 versus AD-induced group. CON, control; O.D, optical density

### Effects of lupeol on the immunoglobulin and cytokine expressions in AD-induced mice

Excessive Th2 response is primarily observed in the acute phase of AD, and mixed Th1/Th2 responses are observed in the chronic phase [32, 33]. Therefore, to confirm whether lupeol affects the Th2 and Th1 responses in AD mice, we measured the levels of IgE and IgG2a in serum using ELISA. Besides, we additionally confirmed the level of DFE-specific IgE which was generated by DFE stimulation. Repeated applications of DNCB/DFE increased the serum levels of all these immunoglobulins. However, lupeol reduced the elevated levels of IgE (total and DFE-specific) and IgG2a, and there were no changes in lupeol only (10 mg/kg) (Fig. 6A).

In AD lesions, cytokines released by inflammatory cells exacerbate inflammation [14, 34]. Hence, we measured the gene expression levels of IL-4, IL-5, and IL-13 as Th2 cytokines, IFN-γ and IL-12 A as Th1 cytokines, and IL-1β as a pro-inflammatory cytokine in ear tissue of AD-induced mice using RT-*q*PCR. The induction of AD-like skin inflammation upregulated all the gene expressions. On the other hand, lupeol reduced the increased gene expressions in a dose-dependent manner (Fig. 6B). Secretion of the representative Th2 cytokine (IL-4), Th1 cytokine (IFN-γ), and pro-inflammatory cytokine (IL-1β) in the ear tissue was also measured using ELISA, and it showed a similar tendency with gene expression (Fig. 6C). In this study, lupeol suppressed the elevated levels of immunoglobulins in serum and cytokines in ear tissue. These results show that lupeol inhibited AD-like skin inflammation by exhibiting anti-inflammatory effects.

## Discussion

AD prevalence has been steadily increasing in recent decades as a result of an increase in harmful environmental factors, affecting approximately 15–20% of children and 1–3% of adults worldwide [1]. The symptoms of AD, including itch, dryness, scaling, and inflamed skin, significantly decrease the quality of their lives [35]. Until now, glucocorticosteroids, calcineurin inhibitors, and antihistamines are the most well-known treatments for AD [36]. Although they temporarily alleviate AD symptoms, long-term use can result in a variety of side effects, including skin atrophy, gastritis, adrenal insufficiency, weight gain, and emotional lability [16]. Given the limitations of currently available drugs, an alternative therapeutic agent for AD is necessary. Natural products, including flavonoids, alkaloids, glycosides, and terpenes display great potential in the treatment of AD with their safety and effectiveness, and they can provide personalized medicine [37, 38]. Specifically, lupeol, a pentacyclic triterpene that exists in fruits, vegetables, and medicinal plants, possesses a wide range of beneficial effects [39]. It has been studied for its anti-inflammatory and anti-allergic properties [20, 21]. Furthermore, lupeol has been found to reduce Th2 immune responses in an asthma [21]. Based on the previous studies, we investigated the applicability of lupeol as a therapeutic agent for AD, also well known as a Th2 cell-associated inflammatory disease.

Keratinocytes, the principal epidermal cells, form physical and functional skin barriers against potential allergens and control immune responses in the skin [9]. Activated keratinocytes produce various pro-inflammatory cytokines (IL-1β and IL-6) as well as chemokines (CCL17 and CCL22) which recruit immune cells into AD lesions and exacerbate the progression of AD [10]. CCL17 and CCL22 have been shown to play a role especially in the migration of Th2 cells reflecting the disease activity of AD [40]. Therefore, regulating the expression of the cytokines and chemokines in keratinocytes could be effective for the treatment of AD. The expressions of inflammatory mediators are regulated by the activation of STAT1, MAPKs, and NF-κB [25–27]. Several studies have indicated that these signaling molecules are critical in the pathogenesis of AD [41–43]. Hence, controlling the activation of the signaling molecules that contribute to the expression of cytokines and chemokines may be related with the alleviation of AD. Lupeol significantly reduced the expression of pro-inflammatory cytokines and chemokines and suppressed the phosphorylation of STAT1, MAPKs (p38 and ERK) and the nuclear translocation of NF-κB in TNF-α/IFN-γ-stimulated keratinocytes. These findings indicated that lupeol presents inhibitory effects on the expression of inflammatory mediators through the blocking of the signaling molecules. By suppressing the activation of keratinocytes, the major epidermal cells actively involved in AD immune responses, lupeol may improve AD-like skin inflammation.

HDMs are the unsurpassed cause of allergic diseases such as AD, asthma, and rhinitis throughout the world, and *Dermatophagoides farinae* used in the experiment is the major species of HDM [8]. Based on the previous studies, DNCB was used as a hapten for pre-sensitization, and DFE was used as an allergen to induce AD-like skin inflammation in this study [44, 45]. Repeated applications of DNCB/DFE showed common phenotypes of AD, including erythema, scaling, hyperplasia, thickened skin as well as infiltration of immune cells. Eosinophils infiltrated into the skin lesions secrete diverse cytokines and chemokines, having immunoregulatory properties [46]. Mast cells are activated by antigen cross-linking with IgE, causing them to release inflammatory mediators such as histamine and cytokines [47]. In our study, lupeol alleviated the phenotypes of AD, including erythema, scaling, and skin thickening. Histological changes such as epidermal and dermal thickening and infiltration of immune cells (eosinophils, mast cells, and CD4^+^ immune cells) were decreased by lupeol in a dose-dependent manner. Overall, these findings indicated that lupeol has a mitigating effect on AD-like phenotypes.

T helper cells which present CD4 glycoprotein on their surface and secrete specific cytokines are important components in AD development, maintenance, and exacerbation [48, 49]. In the acute phase of AD, activated antigen-presenting cells differentiate naïve T cells into Th2 cells that secrete the cytokines like IL-4, IL-5, and IL-13 [11]. IL-4 and IL-13 play a crucial role in immune cell migration and activate IgE class switching from B cells, which sensitizes mast cells [50]. The role of IL-5 in eosinophil recruitment and proliferation is well known [51]. In the chronic phase of AD, Th1 cells also become dominant through IL-12 A induction and secrete IFN-γ [13, 52]. Hence, regulating the Th2 and Th1 responses is a promising therapeutic approach for AD. As mentioned above, the infiltration of CD4^+^ T cells in ear tissue was decreased by lupeol in a dose-dependent manner. Furthermore, lupeol significantly reduced the levels of IgE (total and DFE-specific) and IgG2a in serum as well as the expressions of Th2 and Th1-related cytokines in ear tissue of AD-induced mice. These findings showed that lupeol decreases the Th2 and Th1-mediated immune responses and thus has inhibitory effects in both acute and chronic phases of AD.

Natural products can be excellent alternative therapeutic agents for AD by having safety and providing diverse treatment approaches [37, 38]. In this study, we demonstrated the pharmacological effects on AD of lupeol, a natural product which is abundant in fruits, vegetables, and plants. However, further research is still needed to find the route of administration and formulation which maximize the effects and to confirm the long-term effects of lupeol.

## Conclusions

In this study, lupeol suppressed the expression of inflammatory mediators by blocking STAT1, MAPKs, and NF-κB signaling molecules in activated keratinocytes. The oral administration of lupeol alleviated AD-related phenotypes and inhibited the expression of inflammatory mediators in AD mice. Taken together, we showed the effectiveness of lupeol on AD and suggest lupeol as an alternative therapeutic agent for AD treatment. With technological advances such as oleogel and nanoparticle formulations, we anticipate that those beneficial effects will be further maximized.

## Electronic supplementary material

Below is the link to the electronic supplementary material.

Supplementary Material 1

Supplementary Material 2

## Data Availability

The datasets used and/or analyzed during the current study are available from the corresponding author on reasonable request.

## Abbreviations

AD: Atopic dermatitis
TNF: Tumor necrosis factor
IFN: Interferon
DFE: *Dermatophagoides farinae* extract
Ig: Immunoglobulin
Th: T helper
HDM: House dust mite
IL: Interleukin
CCL: CC chemokine ligand
DMSO: Dimethyl sulfoxide
PBS: Phosphate-buffered saline
DNCB: 2, 4-Dinitrochlorobenzene
Dexa: Dexamethasone
GAPDH: Glyceraldehyde 3-phosphate dehydrogenase
H&E: Hematoxylin and eosin
STAT1: Signal transducer and activator of transcription 1
MAPK: Mitogen-activated protein kinase
NF-κB: Nuclear factor-κB
