# Supplementary material for: Lupeol alleviates atopic dermatitis-like skin inflammation in 2,4-dinitrochlorobenzene/Dermatophagoides farinae extract-induced mice
Source: BMC Pharmacol Toxicol. 2023 Apr 25;24:27. doi: 10.1186/s40360-023-00668-9 (PMC10131421; doi:10.1186/s40360-023-00668-9)
Supplement: Supplementary file 1 — Supplementary Material 1 [file 40360_2023_668_MOESM1_ESM.docx]

**Additional file 1.** Western blots corresponding to Fig. 3.

**
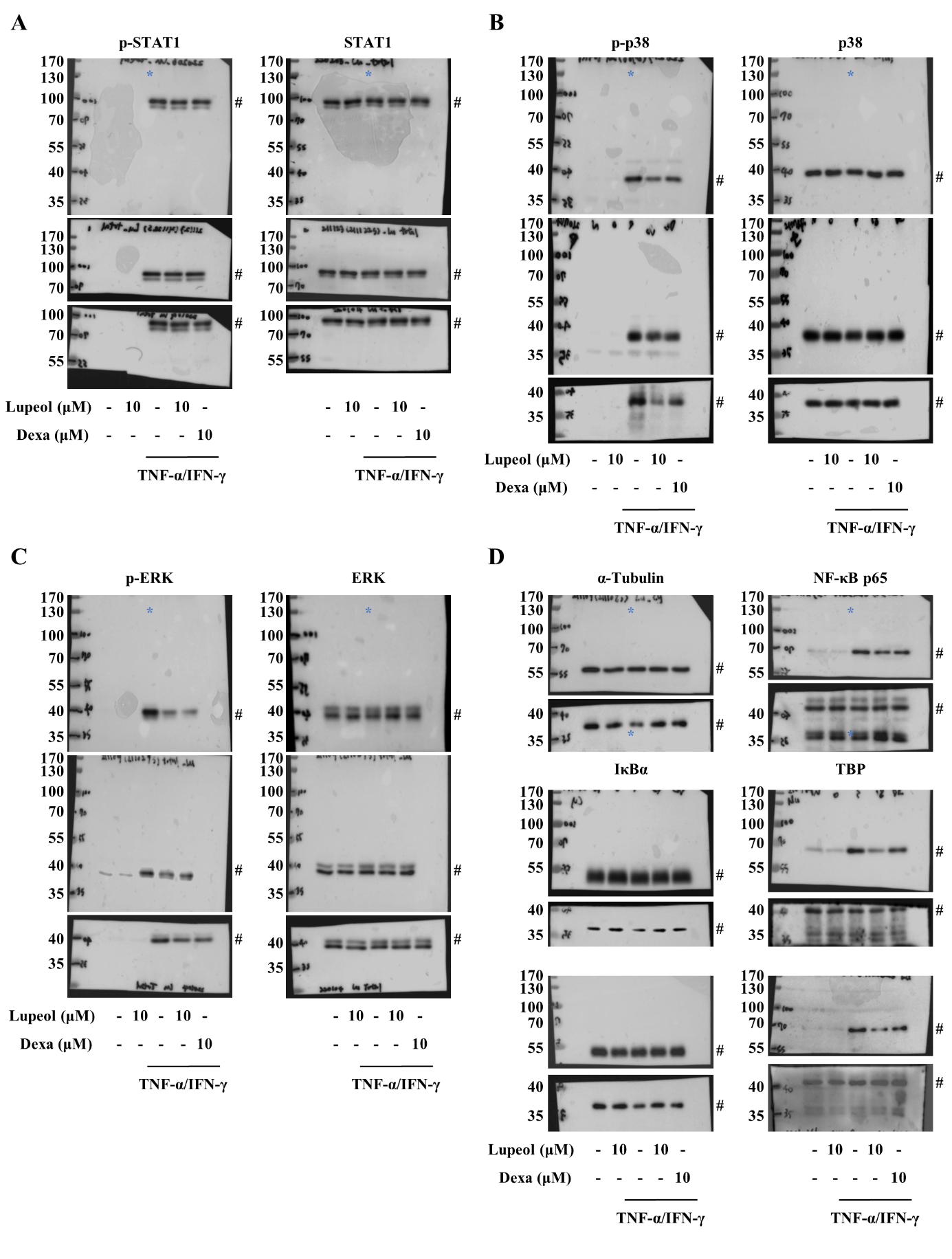
**

(A) Blots of p-STAT1 (84, 91 kDa) and STAT1(84, 91 kDa). (B) Blots of p-p38 (43 kDa) and p38 (40 kDa). (C) Blots of p-ERK (42, 44 kDa) and ERK (42, 44 kDa). (D) Blots of IκBα (37 kDa), α-Tubulin (52 kDa), NF-κB p65 (65 kDa), and TBP (43 kDa). Blots were cut prior to hybridization with antibodies, and full-length images of some replicates are absence. Hash (#) denotes target band. Blots with asterisk (*) were used in main Figure. p-, phosphorylated.
