# Supplementary material for: Lupeol alleviates atopic dermatitis-like skin inflammation in 2,4-dinitrochlorobenzene/Dermatophagoides farinae extract-induced mice
Source: BMC Pharmacol Toxicol. 2023 Apr 25;24:27. doi: 10.1186/s40360-023-00668-9 (PMC10131421; doi:10.1186/s40360-023-00668-9)
Supplement: Supplementary file 2 — Supplementary Material 2 [file 40360_2023_668_MOESM2_ESM.docx]

**Additional file 2.** Effects of lupeol on the cell viability of keratinocytes and toxicity of experimental mice.


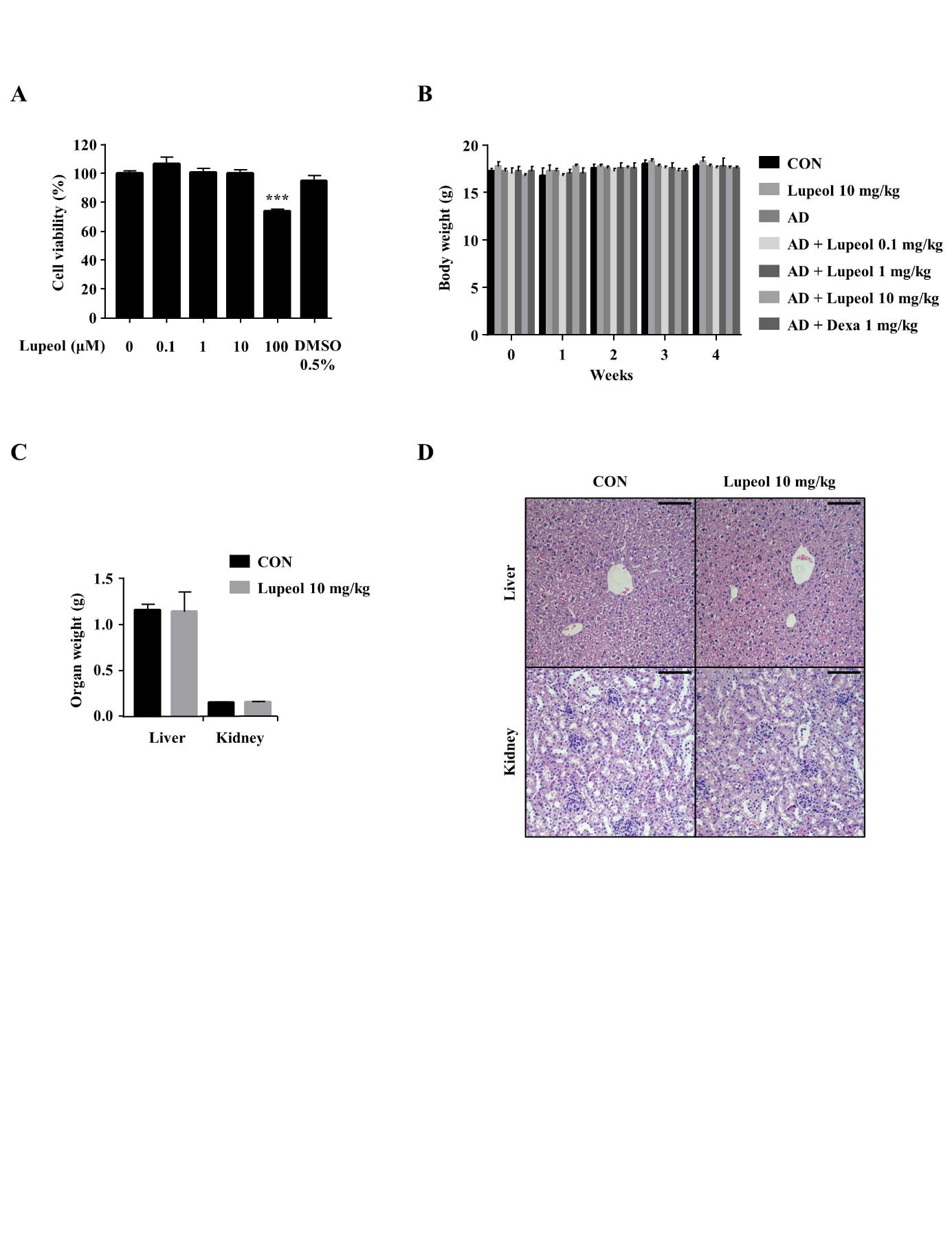


(A) The cell viability of HaCaT cells was verified using MTT assay. HaCaT cells (1 × 10^4^ cells/well in a 96-well plate) were incubated with each concentration of lupeol (0-100 μM) or 0.5% DMSO for 24 h. (B) The body weight was measured on the last day of each week using a portable balance (Ohaus, Parsippany, NJ). (C) The weight of the liver and kidney was measured after they were sacrificed using a PAG214 analytical balance (Ohaus). (D) Representative photomicrographs of H&E-stained organ tissues at ×200 magnification (scale bar: 100 μm). Data are presented as the mean ± SEM (*n* = 5). ****p* < 0.001 versus non-treated group. CON, control.
